# Wearable Continuous Glucose Monitoring and Cognitive Performance in Older Adults With Type 2 Diabetes

**DOI:** 10.1093/geroni/igaf122.1889

**Published:** 2025-12-31

**Authors:** Claire Hoogendoorn, Angel Garcia De La Garza, Nelson Roque, Cuiling Wang, Richard Lipton, Jeffrey Gonzalez

**Affiliations:** Yeshiva University, New York, New York, United States; Albert Einstein College of Medicine, Bronx, New York, United States; The Pennsylvania State University, University Park, Pennsylvania, United States; Albert Einstein College of Medicine, Bronx, New York, United States; Albert Einstein College of Medicine, Bronx, New York, United States; Yeshiva University, New York, New York, United States

## Abstract

Limited work has evaluated continuous glucose monitoring (CGM) in relation to cognitive performance, and this has been conducted largely in type 1 diabetes. We explore associations between CGM and cognition among older adults with type 2 diabetes (T2D). Data are from community-residing adults over age 60 enrolled in the Einstein Aging Study, who self-reported T2D. Participants wore blinded Abbott FreeStyle Libre 3 sensors for 14 days and completed the WAIS III Digit Span, Trails A and B, Multilingual Naming(MINT), and WAIS III Block Design. CGM metrics include mean glucose, variability(coefficient of variation), %time in range(70-180 mg/dL), %time low(<70 mg/dL), %time high(181-250 mg/dL), and %time in very high(>250 mg/dL) glucose. Linear regression models were adjusted for age, sex, education and race/ethnicity. Interim analysis of the first 30 participants(73.9±7.4yrs, 56% female) indicate that they spent 73.8% of their time in range, 16.4% in high, 8.5% in very high, and 1.3% in low glucose. Less time in low glucose was associated with better Block Design scores(β = 0.35,p=0.041). More variability showed a trend with worse MINT scores(β = 0.36,p=0.058) and more time in high showed a trend with slower Trail B performance(β = 0.37,p=0.097). Metrics were not associated with Digit Span or Trail A. A wearable CGM protocol is feasible in older adults and identified clinically relevant metrics that were associated with cognitive performance in T2D. Time in hypo- and hyperglycemia, and variability, were most predictive, with each linked to different cognitive functions including visuospatial processing, language, and executive function. Analyses will be extended to the larger sample.

